# Assessing Readiness and Sustainability for Integrated Care in Ontario, Canada with the Integrated Care Leadership Survey

**DOI:** 10.5334/ijic.7539

**Published:** 2025-08-01

**Authors:** Ruth E. Hall, Kevin Walker, Nusrat S. Nessa, Walter P. Wodchis

**Affiliations:** 1 Health System Performance Network, Institute of Health Policy, Management and Evaluation, University of Toronto, Toronto, Canada; 2 Institute for Better Health, Trillium Health Partners, Mississauga, Canada

**Keywords:** integration, coordinated care, leadership, survey, psychometric analysis

## Abstract

**Introduction:**

Ontario, Canada, is shifting to a more integrated healthcare delivery system through the Ontario Health Team (OHT) initiative. The extent to which OHTs have the capabilities to engage in integrated care is unknown and important to designing implementation supports. This article describes the development and psychometric testing of the Ontario Integrated Care Leadership Survey (OICLS), in 30 OHTs. The OICLS was informed by the Context and Capabilities for Integrated Care framework (CCIC).

**Methods:**

The 42-item survey was distributed electronically to 765 eligible leaders across 30 OHTs; 480 (63%) responded representing approximately 600 organizations. Item analyses and scale psychometric analyses were undertaken to reduce the number of items in the CCIC survey tool while maintaining validity and reliability.

**Results:**

The OICLS survey is comprised of 10 domains covering 12 of 17 capabilities identified in the CCIC. In the total sample, Cronbach’s alpha exceeded 0.7 for nine of the ten domains. Descriptive responses to each of the 39 OICLS closed-ended survey questions illustrate the areas of strength and weakness and where supports are warranted to advance the formation of integrated care delivery systems.

**Conclusion:**

The OICLS offers a brief and valid assessment of foundational aspects of multi-organizational integrated care initiatives.

## Introduction

Integrated care is increasingly becoming a focus for health policymakers as populations are living longer with more chronic conditions necessitating a diverse array of health and social services [1]. The goals of integrated delivery systems are commonly expressed in terms of the Triple Aim (or Quadruple Aim) [2,3]. At present, however, health care in Canada, and many other parts of the world, is still largely siloed with only narrowly focused cross-sectoral collaborative programs. In 2019, the Ministry of Health from Canada’s most populous province, Ontario, launched Ontario Health Teams (OHTs) as a new way of organizing and delivering care where partners, including health and non-health sectors, patients and caregivers come together to design and work as one coordinated team to provide integrated care for their local population [4]. OHTs can be considered a Canadian equivalent to Accountable Care Organizations where a group of providers are clinically and fiscally responsible for the care across the care-continuum for a defined population [4].

The shift to integrated care from previously independent healthcare providers requires considerable reorientation of the mindset and behaviours of organizations and providers [5]. Understanding and measuring the capabilities for integrated care is an important activity in driving health systems toward this goal. Several measurement instruments have been developed to measure the achievement of integrated care delivery (e.g., Framework for Measuring Integrated Patient Care, Development Model for Integrated Care, Provider and Staff Perceptions of Integrated Care, Partnership Self-Assessment Tool) [6,7,8,9]. In the earliest days of development, however, it may be most useful to consider the basic building blocks or capabilities required to support the development of integrated care models [10]. The integrated care literature reveals the success of integrated care is influenced by several organizational and network characteristics such as governance, leadership style, organizational culture, resources, information technology, history of change and innovation, partnering, organizational bureaucracy, commitment to quality improvement, and patient-centeredness [11,12,13,14,15,16,17].

We sought a baseline measurement of the readiness of health system leaders in Ontario to implement integrated care through the OHT initiative. We have named our survey the Ontario Integrated Care Leadership Survey (OICLS) survey. In this paper, we report on the early results and psychometric results of the survey tool created for this purpose.

## Conceptual Framework

**Figure 1:**
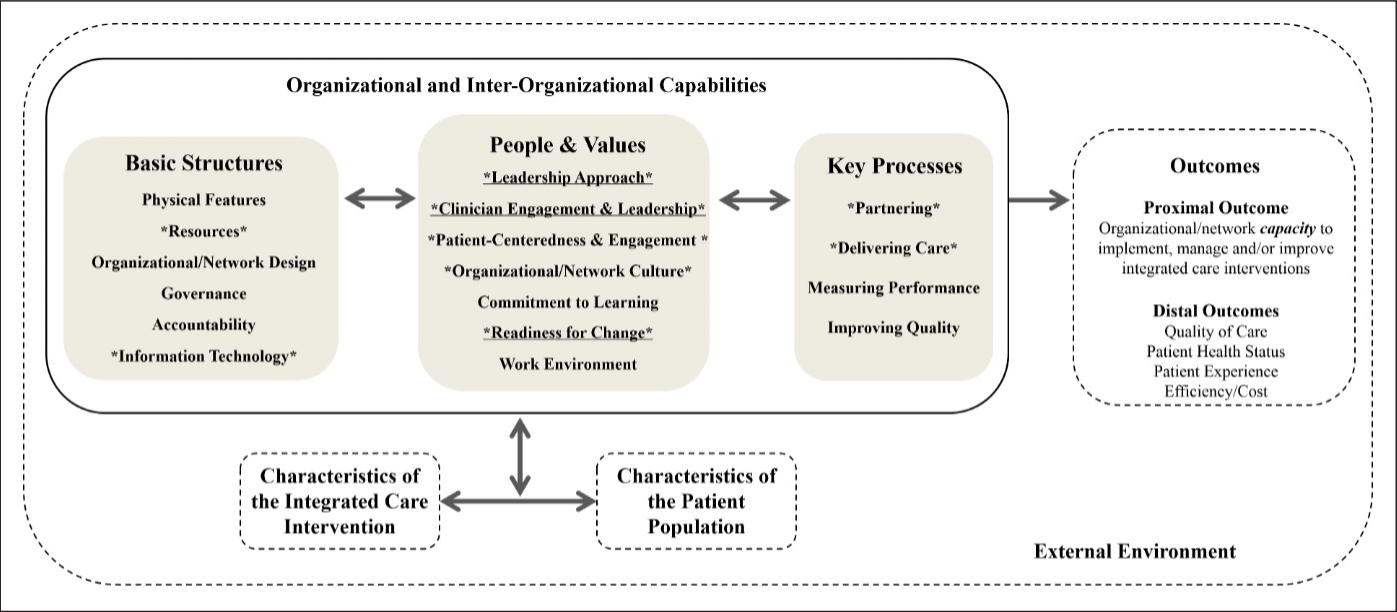
The Context and Capabilities for Integrating Care (CCIC) Framework [18].

The Context and Capabilities for Integrated Care (CCIC) framework was developed for the purpose of measuring the implementation of integrated care [18]. It was developed through a review of the integrated care literature and interviews with leaders and providers engaged in integrated care networks in Ontario context [10,18,19]. It describes key factors, termed contexts and capabilities, that are most important to supporting the implementation of integrated care. These include 17 organizational and network capabilities organized within three categories: 1) Basic Structures; 2) People and Values; 3) Key Processes (see Figure 1) [18]. Interviews with leaders and providers involved in a previous integrated care initiative in Ontario identified nine of the 17 organizational and network capabilities as most essential. These included Basic Structures: i) Resources and ii) Information Technology; People and Values: i) Leadership Approach, ii) Clinician Engagement and Leadership, iii) Patient-Centeredness and Engagement, iv) Network Culture, and v) Readiness for Change; and Key Processes: i) Partnering and ii) Delivering Care [18]. We used the CCIC framework and associated survey tools as our starting point to determine readiness to integrate care among the first cohort of OHTs.

## Description of the Methodology

### Survey Development

In this section, we describe the process undertaken to develop the OICLS survey. The original CCIC framework includes a toolkit with leader and provider surveys to measure 17 context and capabilities for integrated care. The toolkit surveys include items from previously validated tools and scales that had either been used or recommended for evaluating integrated care interventions [12,13]. However, these surveys included over 100 items and we sought to develop a survey with fewer items to reduce respondent burden while maintaining scale validity. We began by narrowing the focus from 17 to the 9 domains of measurement previously identified as most essential for implementing integrated care in Ontario’s context [18]. We removed patient and clinician engagement as the OHT application process required submission of information describing these processes and we determined that a document review would provide sufficient information to assess those essential factors. We reviewed the remaining items for applicability to the context and maturity of OHTs (i.e., at initiation phase of integrating care). Finally, we considered results and learnings from two prior evaluations where the CCIC framework has been applied.

### Item Reduction Analysis

The OICLS survey uses questions from the previously validated surveys included in the CCIC Framework toolkit [18]. In particular, items from the Team Climate Inventory [20], Measure of Network Integration [21], Practice Staff Questionnaire [22], Partnership Self-Assessment Tool [23] and Change Readiness [24] surveys related to aspects of teamwork within and across members, including trust, leadership, partnerships, care coordination, communication and resources were selected [25,26]. Content validity relates to whether the items provide appropriate coverage of the core constructs one intends to measure [27]. Our content validity process went through three rounds.

In the first round, RH and KW independently reviewed the surveys to identify items that were not applicable to the current initiative and design phase [28] of the OHT initiative. These included items that related to evaluating progress and impact. The same two authors then met to discuss their choices and reached consensus on items to be removed. The authors also reviewed factor analysis of the scales from the CCIC survey collected as part of previous integrated care initiatives in Ontario (Health Links and iCOACH) to identify scales with potentially redundant items where deletion would not dramatically decrease the scale’s internal reliability (i.e., Cronbach’s Alpha). Additionally, factor loadings were assessed and, if choosing between items, the items with the higher loadings were retained. After the initial review and analyses, 54 items remained. Authors RH and WPW then met to remove redundant survey items and determine the items aligned with each domain; 42 items were retained. The final version of the survey included 39 substantive items with two to seven items per domain, plus one open ended question and two descriptive items asking about the respondent’s role and type of organization represented.

Table 1 maps the CCIC contexts and capabilities to the corresponding domains measured by the OICLS survey. There are some inter-related concepts that relate to multiple Survey Domains and CCIC capabilities. For example, two OICLS domains, Shared Vision and Roles and Responsibilities, which we classify separately due to their conceptual independence, both measure the CCIC capabilities Partnering and Network Culture. As best possible, we kept the original item phrasing and response options.

**Table 1:** Context and Capabilities for Integrated (CCIC) Framework Domains Captured in the OICLS survey.


OICLS SURVEY DOMAIN	CCIC CAPABILITIES	DESCRIPTION OF CCIC CAPABILITY [18]

1. Leadership Approach (5 items)	Leadership	Methods and behaviours used by formal leaders (vision, respect, trust); strategies to empower members.

2. Shared Vision* (7 items)	Partnering; Network Culture	Development and management of formal/informal connections between organizations (e.g., building common understanding and vision). Widely shared values and habits in the organization.

3. Team Climate (6 items)	Commitment to Learning; Delivering Care; Network Culture	The existence of a set values and practices that support development of new knowledge and insights. Widely shared values and habits in the organization. Methods used by providers in caring for patients.

4. Commitment to Improvement (3 items)	Partnering; Improving Quality; Measuring Performance	Development and management of formal/informal connections between organizations (e.g., building common understanding and vision. Use of practices that continuously improve patient care. The systematic collection of data to measure goal achievement.

5. Readiness for Change – Suitability (6 items)	Readiness for Change	The extent to which organizations and individuals are willing and able to implement change (e.g., attitudes toward change, fit between current strategy and the change)

6. Clinical Functional Integration (2 items)	Delivering Care; Information Technology	Methods used by providers in caring for patients (e.g., use of standardized clinical tools). The availability and ease of use of technology-based communication and information storage mechanisms

7. Roles and Responsibilities* (2 items)	Partnering; Network Culture	Development and management of formal/informal connections between organizations (e.g., implementing referral and discharge/transfer agreements). Widely shared values and habits in the organization.

8. Administration and Management (2 items)	Network Design; Resources	The arrangement of units and roles how they interact; communication and decision-making channels.

9. Financial and Other Material Resources (2 items)	Resources; Information Technology	Availability of tangible and intangible assets for network activities. The availability and ease of use of technology-based communication and information storage mechanisms.

10. Non-financial Resources (4 items)	Resources; Measuring Performance; Improving Quality	Availability of tangible and intangible assets for network activities. Use of practices that continuously improve patient care. The systematic collection of data to measure goal achievement.


*Items in the OICLS Shared Vision and Roles and Responsibilities domains capture both People and Values and Key Processes CCIC categories.

The survey was pre-tested by two individuals involved in separate OHT applications (but who would not be completing the survey) for time, clarity and comprehensiveness. Following the feedback from the pre-testers, minor edits were made to survey items. This included adding response options to the first two items regarding the respondent’s role (e.g., executive, other senior management, service provider) and the type of organization (e.g., acute care hospital, home care, primary care). In addition, the survey was shared with an indigenous-lead OHT to respect the principles of OCAP® (Ownership, Control, Access, and Possession) and minor wording changes (e.g., pluralized communities) were implemented for surveys sent to members of this OHT.

### Study Design, Setting and Survey Administration

The study design was a cross-sectional survey. The survey was distributed to representatives from 30 OHTs that submitted applications to the Ontario Ministry of Health (MOH) to become an OHT in September 2019. At the time of the survey, this constituted the population of OHTs. Twenty-eight more OHTs were subsequently invited to submit full applications between 2020 and 2023. This work is part of a larger study evaluating the implementation of OHTs carried out by the Health System Performance Network and commissioned by the MOH. Ontario is Canada’s most populous province, and the health care system is largely publicly funded and delivered by (mostly non-profit) private health care organizations and providers.

The survey sample population was determined by organizational representatives and individuals (including patient representatives and physicians) who were included as signatories to the OHT application. There were OHTs in both urban and rural settings. Each OHT determined which individuals and organizations would be included as signatories to the application. Study information was sent to a designated evaluation contact for each OHT along with a request for email contact information for all individuals and organizations that signed the OHT application. In the case of organizations, the OHT application rules required that each signatory be able to bind the organization and was therefore most commonly the chair of the board for the organization. As these individuals were often less directly involved in the development of the OHT applications, each signatory organization represented by a board chair was asked to provide the name and email information of the (one) person most involved in the OHT application process.

Data collection was undertaken between December 2019 and March 2020. An invitation was distributed to each individual email including an information letter detailing their rights as participants and a unique link to the online survey, as well as a separate link to opt-out of the survey. A second opportunity to opt-out was offered on the introduction page of the survey.

#### Participants and Data Collection

Participants provided informed consent by completing the self-completed web-based survey available through a secure Canadian survey platform. Up to four reminders were sent via email to non-responders over a six-week period. However, due to delays with some teams, data collection continued with these teams until mid-March 2020. Additionally, OHT evaluation contacts were asked to encourage their members’ participation if their OHT’s response rate was <50% or if there were fewer than six responses after three reminders. The survey was only available in English at the time of distribution. All substantive items were optional, but most items did not have a *Not Applicable* or *Don’t know* option. If respondents left a question blank, they were alerted before moving to the next page, but were not required to respond in order to continue completing the survey. The study was approved by the Research Ethics Board of the University of Toronto.

### Measurement

Five-point Likert response scales (e.g., Strongly Disagree = 1, Disagree = 2, Neither Disagree or Agree = 3, Agree = 4, and Strongly Agree = 5, Poor = 1, Fair = 2, Good = 3, Very Good = 4, Excellent = 5, None of what it needs = 1, Almost none of what it needs = 2, Some of what it needs = 3, Most of what it needs = 4, All of what it needs = 5 and Not at all = 1, Minimally = 2, Somewhat = 3, Mostly = 4, Completely = 5) was used for all substantive items, except three questions, one, which had Strongly Agree = 1, Agree = 2, Neither Disagree or Agree = 3, Disagree = 4, Strongly Disagree = 5, the second which asked the respondent to describe their organization’s attitude toward change (resistant, cautious, open, innovative), and the third, which was open-ended. The ten-domain survey (42 items) typically took less than 15 minutes to complete (median 9 minutes).

### Statistical Analysis

Likert response options were scored from 1–5, where a higher score indicated a more favourable response, with exception of question 38. Item analysis included an assessment of the number of respondents, the frequency and mean of the item response and the proportion of responses in the top and lowest categories. Internal consistency of the ten domains was considered using Cronbach’s alpha [29]. Cronbach’s alpha provides a measure of the internal consistency of a test or scale and should be determined before a test can be employed for research or examination purposes to ensure validity [30]. Psychometric analysis was performed on each domain to ensure that items comprising each pre-defined domain continued to load together in the sample of OHT representatives.

Each question was identified with one domain even though there may be conceptual and statistical overlap in some cases. Due to missing values, we used the expectation-maximization algorithm to estimate the covariance matrix for the items comprising each scale. For each scale separately, we extracted a single factor and kept all items with an absolute value of the factor loading greater than 0.4. Cronbach’s alpha was then calculated using the remaining items in each scale to test for internal reliability. Opportunities to improve internal reliability was determined by dropping one item at a time, but if reliability of the original scale was sufficient (α > 0.7), maintaining the integrity of the scale by retaining survey items took precedence over small improvements in reliability.

Although the primary purpose of this study was to report on the internal reliability of the OICLS survey, we also report on the overall scores for the full sample to enable comparisons to related integrated care studies. Detailed OHT-specific results are available elsewhere [31].

## Results

### Respondents

The evaluation team received contact details for 765 individuals; the mean number of individuals identified as signatories to the application for each OHT was 26 with a range of 6 to 142. After screening for staff roles and government representatives, the electronic OICLS questionnaire was sent to 745 potential participants from 30 OHTs. Following repeated emails, 480 people from 30 OHTs responded (range 6 to 47 per OHT). Among respondents, 79.8% were administrators, 14.8% had clinical roles (Table 2).

**Table 2:** OHT Survey Respondent Characteristics (N = 480).


CHARACTERISTIC	N	%

**OHT location**		

Urban/Suburban	408	85%

Small Community/Rural*	72	15%

**Current Role** [select one]		

Chief Executive Officer, President or Executive Director	257	53.5

Other Senior Management (COO, CFO, Vice President, Chief of Staff)	68	14.2

Administrator, General Manager, Director of Care	58	12.1

Physician or Other Clinical Role	71	14.8

Patient/Caregiver	15	3.1

Other	11	2.3

**Type of Organization Represented** [select all applicable]		

Community Support Services (Including Community Mental Health & Addictions)	176	36.7

Primary Health Care Practice	149	31.0

Hospital		

- Acute Care Inpatient Hospital	39	8.1

- Mental Health Inpatient Hospital	6	1.3

- Rehabilitation or Complex Continuing Care Hospital	14	2.9

Long-Term Care	54	11.3

Home Care	72	15.0

Public Health	13	2.7

Patient and Family Advisory Council	16	3.3

Other^§^	77	16.0


* A Small community/rural OHT was defined as populations <150,000. § Examples of other types of organizations represented include municipalities, paramedic services, hospices, shared (digital) services organizations.

### Respondent Characteristics

Among the 30 teams in this first round of applications to become OHTs, 19 were from urban/suburban settings (populations >150,000) and 11 in small community/rural settings (populations <150,000 population). Additionally, 19 were deemed to be hospital-led applications and the remaining non-hospital led. Location and lead organization of each OHT was obtained from the OHT application forms (see Table 2 for respondent characteristics).

Most survey respondents (53.5%) were in executive leadership roles (e.g., Chief Executive Officers, Presidents and Executive Directors). Approximately 26% of respondents were in senior management (e.g., Vice President) or director or managerial roles. Fifteen percent were clinicians with most being physicians. There was a small number of patients and caregivers and other roles noted (e.g., board member, municipal councillor, community representative). The most common sector of survey respondents was community support organization (36.7%) followed by primary care practices (31.0%). Home care and long-term care organizations comprised 15.0% and 11.3% of the survey respondents respectively.

Of the 765 individuals emailed an invitation to the OICLS survey, 480 submitted their survey for an overall response rate of 63%. The mean completion rate across the 480 respondents was 98.1%. The mean percentage of missing values across survey items was 1.7%, with a range of 0% to 10.7%. The highest number of missing values was for question 29, which asked about the sufficiency of financial (money) resources available to the OHT.

### OICLS Items Characteristics and Factor Loadings

Table 3 presents the 34 out of 39 OICLS items and response characteristics grouped according to the ten domains. There were five items that did not load sufficiently on the existing factors and did not have support for inclusion as distinct domains.

**Table 3:** Ontario Integrated Care Leadership Survey (OICLS) questions and response characteristics (N = 480).


OICLS QUESTIONS INCLUDED IN THE 10 DOMAINS**			RESPONSE OPTIONS		

	n (%)	MEAN (SD)	OPTION 1 (%)	OPTION 5 (%)	OVERALL CRONBACH ALPHA

Shared Vision* By working together, how well, at present, are the members in your [program name] able to					0.893

3. Develop goals that are widely understood and supported among members	479 (99.8)	3.873 (0.807)	0.6	21.5	

4. Identify how different organizations/programs in the community could help	478 (99.6)	3.688 (0.812)	0.6	14.6	

5. Respond to the needs and problems of the community	477 (99.4)	3.669 (0.783)	0.2	12.8	

6. Include the views and priorities of the people affected by the OHT’s work	478 (99.6)	3.692 (0.880)	1	16.3	

7. Obtain support from individuals and organizations in the community	477 (99.4)	3.692 (0.837)	0.8	14.9	

**Leadership Approach§** Please rate the total effectiveness of your [program name] leadership in each of the following areas					0.945

18. Empowering people/members involved in the OHT.	479 (99.8)	3.793 (1.007)	2.3	26.7	

19. Communicating the vision of the OHT.	479 (99.8)	3.754 (1.068)	2.3	29	

20. Creating an environment where differences of opinion can be voiced	479 (99.8)	3.814 (1.102)	3.3	33.6	

21. Helping the OHT to be creative and look at things differently.	479 (99.8)	3.668 (1.077)	2.7	25.3	

22. Fostering respect, trust and inclusiveness amongst OHT members.	479 (99.8)	3.889 (1.118)	3.5	37.6	

Team Climate* At present in this [program name]					0.902

15. We are prepared to question the basis of what the team is doing	477 (99.4)	3.964 (0.979)	1.9	35	

16. We critically appraise potential weaknesses in what our OHT is planning	478 (99.6)	3.785 (0.987)	1.7	26.2	

17. The members of the OHT build on each other’s ideas	479 (99.8)	4.127 (0.939)	1	43.8	

39. We have a ‘we are in it together’ attitude	479 (99.8)	4.267 (0.948)	1.7	53.2	

40. We take the time needed to develop new ideas	479 (99.8)	3.954 (0.947)	1.7	32.8	

41. To what extent do you think your OHT’s objectives can actually be achieved	479 (99.8)	3.887 (0.795)	0	22.1	

Clinical Functional Integration* At present in this [program name]					0.805

12. We share tools for clinical coordination	477 (99.4)	3.264 (0.999)	3.4	10.9	

13. We share clinical information across partners	477 (99.4)	3.195 (1.001)	3.8	9.6	

Readiness for Change – Suitability* To what extent do you agree with the following statements?					0.758

34. I think that my organization/practice setting will benefit from this change	464 (96.7)	4.218 (0.940)	1.7	48.1	

35. This change will make my role easier	450 (93.8)	3.049 (1.124)	4.4	11.8	

36. I feel it is worthwhile for me that the organization adopted this change	465 (96.9)	4.353 (0.876)	1.5	55.5	

Commitment to Improvement* At present in this [program name]					0.764

8. We have a common vision of how to improve the integration of care	479 (99.8)	4.163 (0.862)	0.6	40.9	

11. We have agreed to share responsibility for achieving improved patient outcomes	479 (99.8)	4.161 (0.911)	1.3	43.4	

14. We have used data to identify the improvements for our target populations	479 (99.8)	3.825 (0.965)	1.3	26.9	

Roles and Responsibilities* At present in this [program name]					0.881

9. We understand the role we will play in taking responsibility for the local population	479 (99.8)	3.942 (0.915)	0.8	29.6	

10. We understand the role we will play in coordinating care	479 (99.8)	3.764 (0.933)	0.6	23.4	

**Administration and Management§** Please rate the effectiveness of your [program name] in carrying out the following activities					0.898

23. Communicating among members	480 (100)	3.823 (1.036)	1.9	30.2	

24. Organizing OHT member activities, including meetings and projects	480 (100)	4.044 (1.012)	1.9	40.4	

**Financial and Other Material Resources¥** For each of the following types of resources, to what extent does your OHT have what it needs to work effectively					0.683

29. Money	427 (89)	2.440 (0.871)	16.4	0.7	

30. Tools and technologies	457 (95.2)	2.823 (0.823)	5.5	2.4	

**Non-financial Resources¥** For each of the following types of resources, to what extent does your OHT have what it needs to work effectively					0.803

25. Skills and expertise	468 (97.5)	3.703 (0.692)	0.6	9.4	

26. Data and information	462 (96.3)	3.381 (0.726)	0.9	5.8	

27. Ability to identify target population criteria and deliver interventions	456 (95)	3.697 (0.768)	0.7	13.6	

28. Connections to political decision-makers, government agencies	436 (90.8)	3.612 (0.873)	1.4	16.1	


* A 5-point Likert response scale where Strongly Disagree = 1, Disagree = 2, Neither Disagree or Agree = 3, Agree = 4, and Strongly Agree = 5. § A 5-point Likert response scale where Poor = 1, Fair = 2, Good = 3, Very Good = 4, Excellent = 5. ¥ A 5-point Likert response scale where None of what it needs = 1, Almost none of what it needs = 2, Some of what it needs = 3, Most of what it needs = 4, All of what it needs = 5. £ A 5-point Likert response scale where Not at all = 1, Minimally = 2, Somewhat = 3, Mostly = 4, Completely = 5. ** There were five items that were not included into the domains. These items were as follows: 31) Organization or practice setting’s attitude toward change: [Resistant to change | Cautious toward change | Open to change | Innovative] To what extent do you agree with the following statements:* 37) I have the skills that are needed to make this change work 38) This change will disrupt many of the working relationships I have developed 32) Your organization’s shared values are compatible with those of other OHT members 33) Your organization’s staff have a strong sense of belonging to your OHT

The number of responses for each item is presented along with the mean and the proportion of responses in the top and lowest categories to assess floor and ceiling effects. Overall, respondents tended to rate positively with each of the 34 statements; of the 480 respondents the mean score across all items was 3.74/5.0. The proportion of highly favourable (a value of 5) responses ranged from 0.7% for having the money needed to work effectively to 55.5% reporting they felt it was worthwhile for their organization to be part of this change. The proportion of least favourable (a value of 1) responses ranged from 0% reporting little ability the OHT’s objectives could be met achievable to 16.4% not having the money needed to work effectively.

Table 3 also presents the reliability analysis for each of the domains in our survey. Each of the factor scores were analyzed independently for each domain. Cronbach’s alpha showed the survey domains reached above acceptable reliability, α ≥ 0.75, [32] for all but the two-item Resources domain. Most items appeared worthy of retention, resulting in a decrease in the alpha if deleted. An exception to this was in the Readiness for Change domain, which would have increased to α = 0.83 with removal of the item “this change will make my role easier”. ‘Use of data to identify improvements’ was also a slightly dissonant item. All factor loadings were well above the customary 0.40 loading threshold (data not shown).

## Summary Results

**Figure 2:**
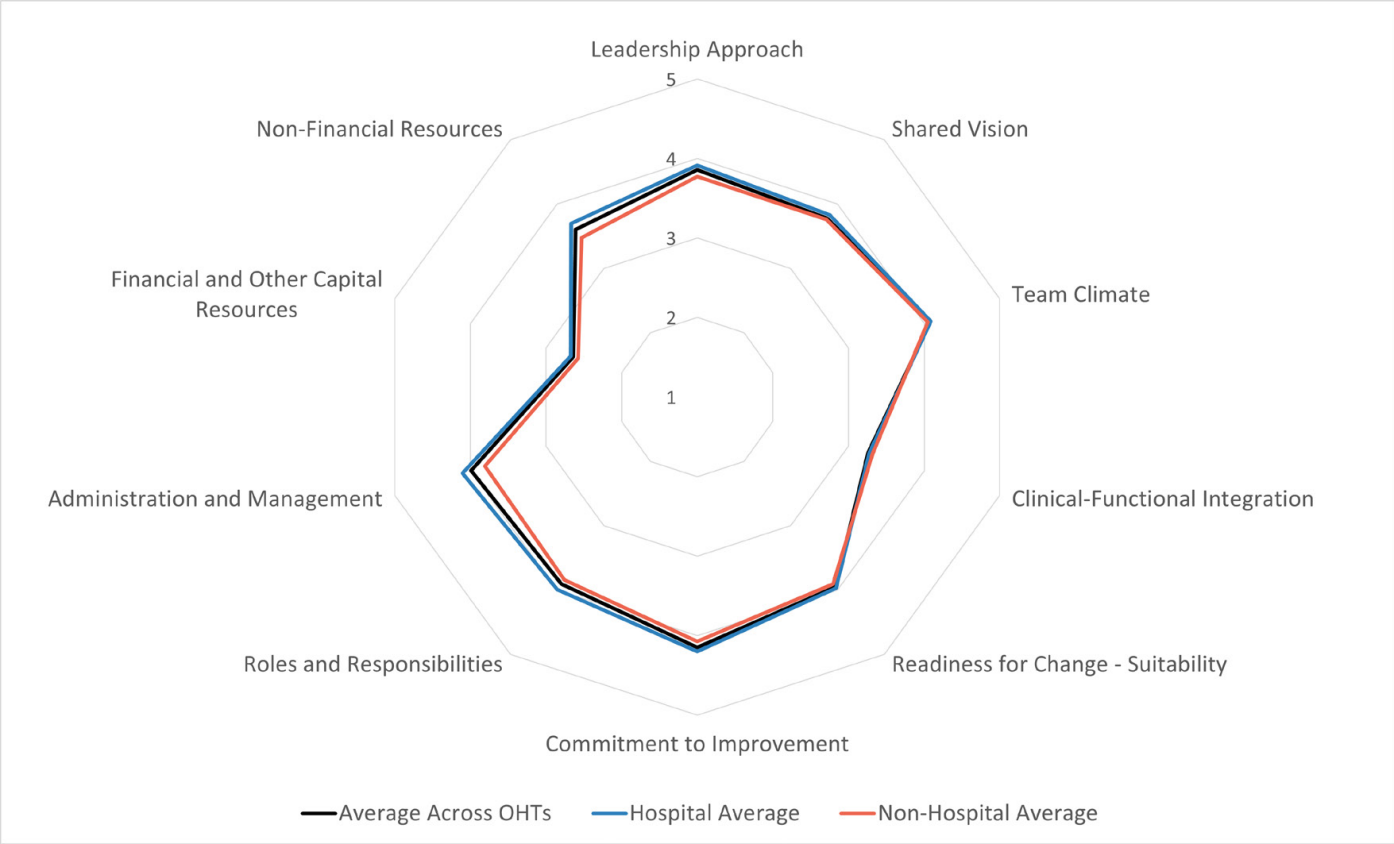
OHT Summary Results on 10 Domains of the Ontario Integrated Care Leadership Survey by Leadership Organization.

The results of each of the scales across the participating respondents are shown in Figure 2. For this figure, we stratified the results according to the leading organization for each OHT. In general, all OHTs had the strongest capabilities in the domains of Commitment to Quality Improvement, Administration and Management, and Team Climate, each of which had an average above 4.0 for Hospital-led OHTs. The lowest scores were for Financial Resources, Clinical Functional Integration and Non-Financial resources, each of which had scores below 3.5 amongst the Non-hospital-led OHTs. Otherwise, all scores lay between 3.5 and 4.0 for both Hospital and Non-hospital-led OHTs. Only Administration and Management and Non-Financial Resources domains were scored significantly higher by respondents for Hospital as compared to Non-hospital-led respondents (t-test p < 0.01 for each).

## Discussion

The OICLS survey was developed for a more concise instrument to assess key capabilities needed for integrated care initiatives, with an emphasis on leadership, partnerships, delivering care, and resources. Furthermore, the OICLS survey was tested in 30 integrated care teams (OHTs) in the initial stages in their evolution to maturity across teams that were Hospital- and Non-hospital-led in the Ontario. The OICLS survey scales align to the majority of the capabilities identified in the CCIC framework and appear to be an internally consistent measure among the leaders involved in the early stages of forming OHTs. The 465 OHT leaders and 15 patients/caregivers who completed the OICLS had a range of roles and came from both urban and rural regions. There were few missing responses (<2%) to any of the 39 OICLS questions supporting the acceptability of questions to respondents.

Our analyses confirmed nine of the ten scales had acceptable levels of internal consistency (Cronbach’s α > 0.75). Item analyses indicate that all questions were strongly correlated with their scales based on the CCIC Framework with exception of the Financial and Other Material Resources domain. However, when we combined the all the items from the Financial and Non-Financial Resource domains (i.e., created a Resource domain) the alpha increased to 0.817 (data not shown). The distinction from a funding and organizational capability perspective led us to retain these as separate domains.

Using the CCIC framework to measure key contexts and capabilities supporting integrated care delivery early in the OHT development allows for an assessment of “readiness to integrate” and the development of targeted change management strategies that address problem areas as OHTs embark on delivering patient-centred, high quality coordinated and integrated care. Minkman argues integrated care initiatives begin with an initiation and design phase, proceed to the execution and experimentation phase, followed by expansion and monitoring, and finally, at maturity where there is consolidation and transformation [28]. Minkman’s framework also has an accompanying survey that can be used to identify the developmental stage of integrated care initiatives [28]. At the time of this analysis, OHTs were just applying to obtain sanction to develop this new model of care and all were in the initiation and design phase. The adapted CCIC survey was designed to capture the context and capabilities of OHTs in the first phase of their development. Future research will investigate whether the OICLS survey is sensitive to changes over time, as OHTs begin to implement their integrated care plans across the care continuum for their attributed populations.

The relative strengths amongst OHTs across domains are also comparable to results reported by studies that used the SCIROCCO assessment tool. Although the measures are not fully comparable, we mapped the definitions of the 12 SCIROCCO domains defined by Lim et al [34] to the definitions from the CCIC shown in Table 1. Based on scores reported for integrated care initiatives in Singapore, Basque (Spain), Scotland and Puglia (Italy), the SCIROCCO tool similarly reported highest scores for Breadth of Ambition (Commitment to Improvement in the OICLS), Readiness for Change (same in OICLS), and the lowest scores for Funding (OICLS Financial Resources) similar to our findings for Ontario. Ontario scored relatively higher in the Coordination of Care.

We reported the OHT overall scores separately for Hospital-led and Non-hospital-led OHTs. Prior studies of Accountable Care Organizations (ACOs) in the United States have shown that, in the early days of development, that ACOs led by physician organizations had performance than hospital-led organizations [33]. In contrast, the OICLS found that hospital-led OHTs had greater capability as compared to Non-hospital-led OHTs. It remains to be seen whether these capabilities translate to overall health system performance improvement.

The original public report on individual OHT results [31] also showed considerable differences in OICLS results across individual OHTs with an indication of discriminative validity between OHTs. It remains to be seen whether such differences are sustained and whether the OICLS is sensitive to changes in OHT capabilities over time.

### Limitations

There are several limitations to this research. First, this survey was developed to assess readiness for integrating care among the several provider organizations within a publicly-funded siloed healthcare system which may limit the generalizability to other jurisdictions and contexts. Second, because this survey has not been administered elsewhere, we do not have normative values for the domain results. Although we had access to CCIC data from prior examples in Ontario for domain reliability analyses, these data were based on samples of less than 100 respondents in each instance and have been sparsely reported and not in peer-reviewed literature. Third, although physician engagement and patient involvement are considered a priority for integrating care, we intentionally did not include domains/items to measure the extent of the engagement/centredness as we determined the information extracted from the OHT applications would enable us to evaluate physician/clinical and patient engagement/centredness and we wished to minimize survey respondent burden [35]. Adding related items should be considered when other sources of data are not available. As these measures were also lacking from the CCIC survey tools, an update to that framework should be considered as well.

Despite these limitations, the response rate for this study was high compared to response rates to surveys among health system leaders and providers and allowed us to conduct reliability analysis which suggests our survey has acceptable reliability for factor analyses [36].

## Conclusion

The Ontario Integrated Care Leadership Survey results capture the initiation and design phase of Ontario’s journey to transforming from siloed to integrated care. The CCIC framework was the foundation for the domains and items in our survey and results show we maintained domain integrity while reducing respondent burden. It will be important to re-assess the OHTs on many of these domains to determine whether beliefs, attitudes and commitments are sustained as OHTs begin to implement their integrated care plans.

This shortened version of multiple survey instruments included in the CCIC toolkit may be useful to others looking to examine “readiness” for integrated care and to determine if the strengths and challenges we observed among multi-organization partnerships are similar across jurisdictions. The use of this survey amongst managers and providers as OHTs begin to implement new plans is another opportunity for new knowledge and comparative information. Examining relationships between the OICLS scores and provider experience (e.g., job satisfaction, burnout) may add to our knowledge of positive and negative provider experiences associated with integrated care delivery. These activities are supported by the full publication of the survey items in Table 2 of this manuscript.
